# Small Molecules Incorporating Privileged Amidine Moiety as Potential Hits Combating Antibiotic-Resistant Bacteria

**DOI:** 10.3390/ph16071040

**Published:** 2023-07-22

**Authors:** Selwan M. El-Sayed, Samar A. Ahmed, Kanika Gulia, Justin R. Lenhard, Ahmed H.E. Hassan, Abdelbasset A. Farahat

**Affiliations:** 1 Department of Medicinal Chemistry, Faculty of Pharmacy, Mansoura University, Mansoura 35516, Egypt; salwanmahmoud@mans.edu.eg; 2 Pharmaceutical Chemistry Department, Faculty of Pharmacy, Delta University for Science and Technology, Gamasa 35712, Egypt; 3 Department of Clinical and Administrative Sciences, College of Pharmacy, California Northstate University, Elk Grove, CA 95757, USA; samar.ahmed@cnsuedu.onmicrosoft.com (S.A.A.); justin.lenhard@cnsu.edu (J.R.L.); 4 Master of Pharmaceutical Sciences Program, California Northstate University, 9700 W Taron Dr., Elk Grove, CA 95757, USA; kanika.gulia7488@cnsu.edu; 5 Medicinal Chemistry Laboratory, College of Pharmacy, Kyung Hee University, Seoul 02447, Republic of Korea; 6 Department of Pharmaceutical Organic Chemistry, Faculty of Pharmacy, Mansoura University, Mansoura 35516, Egypt

**Keywords:** antibacterial agents, diamidines, microbial resistance

## Abstract

The continuing need for the discovery of potent antibacterial agents against antibiotic-resistant pathogens is the driving force for many researchers to design and develop such agents. Herein, we report the design, synthesis, and biological evaluation of amidine derivatives as new antibacterial agents. Compound **13d** was the most active in this study against a wide range of antibiotic-resistant, and susceptible, Gram-positive, and Gram-negative bacterial strains. Time–kill assay experiments indicated that compound **13d** was an effective bactericidal compound against the tested organisms at the log-phase of bacterial growth. Docking simulations were performed to assess *in silico* its mode of action regarding UPPS, KARI, and DNA as potential bacterial targets. Results unveiled the importance of structural features of compound **13d** in its biological activity including central thiophene ring equipped with left and right pyrrolo[2,3-*b*]pyridine and phenyl moieties and two terminal amidines cyclized into 4,5-dihydro-1*H*-imidazol-2-yl functionalities. Collectively, compound **13d** represents a possible hit for future development of potent antibacterial agents.

## 1. Introduction

Penicillin discovery and the subsequent development of several antibacterial chemotherapeutics over the 1950s to 1970s marked the ‘golden antibiotic era’ [1]. Nealy half of the known antibiotics were discovered and developed during these decades, which significantly reduced morbidity and mortality from bacterial infections. Unfortunately, the inappropriate, irresponsible, and excessive use of antibiotics resulted in the emergence and development of antimicrobial resistance (AMR) that impaired the effectiveness of antimicrobial treatment protocols [2]. Such a situation raises alarms of entry into a ‘post-antibiotic era’ in which AMR will render minor injuries and infections fatal [3]. Around 5 million deaths because of AMR were estimated in 2019 including nearly 1.3 million deaths because of bacterial AMR [4]. The global toll of AMR deaths is estimated to increase to 10 million deaths annually by 2050 [5]. It is therefore urgent to develop new agents effective against antibiotic-resistant bacteria.

As a workplan is needed to combat antimicrobial resistance, the World Health Organization (WHO) announced a global priority list of drug-resistant species that are the most urgent to be considered for development of new agents [6]. In the list, the ESKAPE bacteria that include multidrug-resistant *Enterococcus faecium*, *Staphylococcus aureus*, *Klebsiella pneumoniae*, *Acinetobacter baumannii*, *Pseudomonas aeruginosa*, and *Enterobacter* spp., were listed as critical and high priority organisms. Such ESKAPE organisms are a global leading cause of serious, life-threatening infections [7,8,9]. In many cases, ESKAPE pathogens are a leading cause of dangerous nosocomial infections resulting in deaths, particularly in immunocompromised patients [10]. The ability of such antibiotic-resistant bacteria to overcome the action of current drugs might be attributed to drug inactivation, modifications of the drug’s target receptor, or decreased drug accumulation within the organism either by efflux mechanisms or via decreased permeability. To foil bacterial resistance, it may be beneficial not only to address new targets but also to develop multitarget agents [11,12,13,14].

Selective toxicity is a desirable key property for the successful development of antimicrobial agents. Molecules that act on bacterial-specific targets could be toxic to bacteria but non-toxic for mammalian cells. Consequently, molecules targeting bacterial cell wall biosynthesis can selectively kill bacteria but do not impair mammalian cells. Bacterial undecaprenyl diphosphate synthase (UPPS) is an enzyme absent in mammals that is essential for the bacterial isoprenoid biosynthetic pathway of cell wall synthesis [15]. Consequently, UPPS could serve as a new promising target for the development of novel antibacterial agents. Ketol-acid reductoisomerase (KARI) is another mammals-absent key bacterial enzyme that can serve as a promising molecular target for developing novel antibacterial agents. KARI is involved in the biosynthesis of branched-chain amino acids (BCAA), which are the essential leucine, valine, and isoleucine amino acids. In contrast to bacteria and other microbes that utilize KARI for synthesis of such essential BCAA, mammals have no KARI. Accordingly, KARI inhibitors could be safe and effective antibacterial agents [16,17,18].

Minor groove binders (MGBs) are small molecules that bind within the minor groove of DNA B-form. Such binding disrupts nucleosomes resulting in suppression of DNA replication and transcription, which are essential biological processes for bacterial growth and multiplication. Such DNA replication and transcription requires promoter sequences that are sequences at which DNA unwinding is initiated. Such promoters involve either AT-rich or GC-rich repeated sequences. While human promoter sequences are mainly GC-rich, microbial promoter sequences predominantly involve AT-rich sequences [19,20,21]. Furthermore, B-DNA form of AT-rich sequences feature a narrower, deeper minor groove which is capable of establishing more hydrogen bond interactions than GC-rich sequences [22,23,24]. Such facts might offer an opportunity to develop selective and safe antimicrobials.

Diamidines are an interesting class of compounds that reported to possess interesting bioactivities [25,26,27,28]. While the literature reports show that some diamidines have antibacterial activities, studies of their antibacterial effects against antibiotic resistant bacteria, to the best of our knowledge, are scant and incomplete [29,30,31]. Interestingly, it was recently discovered that diamidines such as pentamidine and its congeners sensitize antibiotic resistant Gram-negative bacteria to Gram-positive antibiotics [32,33]. It might be intriguing to design and more thoroughly investigate the direct antibacterial effects of novel diamidines against antibiotic-resistant bacteria in comparison with antibiotic-susceptible bacteria. Consequently, we addressed, herein, the design, synthesis and assessment of small molecules incorporating the privileged amidine moiety to discover new multitarget antibacterial hit compounds against antibiotic-resistant bacteria. In this report, we present our approach and interesting results.

## 2. Results and Discussion

### 2.1. Design Approach

**Figure 1:**
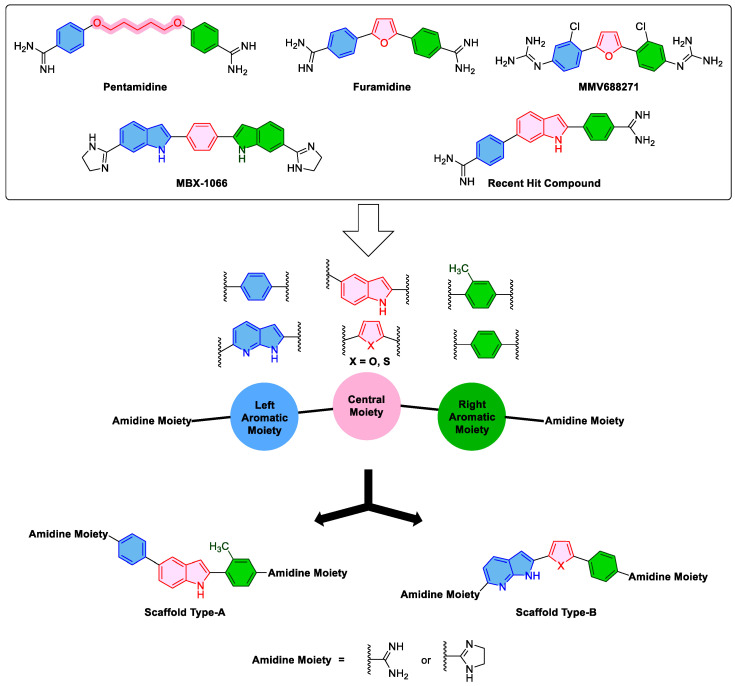
The literature -reported compounds and the design of the new targeted compounds.

The structure of diamidines involves a central moiety linked to left and right aromatic moieties (Figure 1). Both left and right aromatic moieties carry an amidine moiety that might be in the form of an acyclic or cyclic moiety. Amongst interesting diamidines, the antiparasitic drug pentamidine (Figure 1) was found not only as a potential MGB but also as an inhibitor of *E. coli* KARI [34,35,36,37,38]. While pentamidine has a flexible central moiety of pentamidine, the rigid analog, furamidine has potential antimicrobial activity via MGB [34,37]. Compound MMV688271 (Figure 1), a furamidine analog which has chlorophenyl left and right aromatic moieties each bearing the amidine-like biguanide fragment, was found as an effective inhibitor of *Mycobacterium tuberculosis* KARI [18]. Compound MBX-1066 (Figure 1), which has larger left and right aromatic indole moieties combined with cyclic amidine moiety, was identified as an antibacterial agent eliciting potential UPPS inhibition that impairs bacterial cell wall synthesis in addition to its potential MGB activity [38,39]. Interestingly, a recently identified hit compound (Figure 1) possessing central indole ring combined with left and right phenyl rings each bearing acyclic amidine moiety was reported to exhibit potential activity against Gram-positive antibiotic-resistant ESKAPE bacteria [6]. However, the compound showed lower activity against Gram-negative antibiotic-resistant ESKAPE bacteria.

Molecular hybridization is well-acknowledged drug strategy that can afford bioactive multitarget compounds [40]. Aiming to discover new multitarget antibacterial agents against antibiotic-resistant ESKAPE pathogens and considering the diverse structural features of the literature-reported compounds shown in Figure 1 coupled with their antibacterial activity mediated through UPPS or KARI inhibition and MGB, new hybrid compounds were designed. As illustrated in Figure 1, designed compounds possessing scaffold A were planned to incorporate the larger central indole ring connected to left and right phenyl rings at 2- and 5-positions, each of which bears at *para* position either acyclic unsubstituted amidine moiety or 4,5-dihydro-1*H*-imidazol-2-yl as cyclic amidine moiety inherited from compound MBX-1066. Such design of scaffold A might be in part a positional isomer to the recently identified hit compound (Figure 1) in which the left aromatic ring was translocated from 6- to 5-position of the indole ring. The right phenyl ring connected to 2-position of the central indole ring was planned to bear a methyl substituent as an isosteric replacement for the chloro-substituent in compound MMV688271. Meanwhile, designed compounds conforming to scaffold B have the smaller five membered furan or thiophene rings as the central moiety connected to a left fused bicyclic pyrrolo[2,3-*b*]pyridine fragment as an isostere to the left indole ring of compound MBX-1066. Concurrently, the right indole ring of compound MBX-1066 was replaced in scaffold B with a phenyl ring inherited from other known compounds shown in Figure 1. Finally, the cyclic amidine moiety of compound MBX-1066 was maintained or replaced by acyclic unsubstituted amidine moiety. Such designed diverse structures of scaffolds A and B were synthesized and biologically evaluated for antibacterial activity with a particular consideration to antibiotic-resistant ESKAPE bacteria.

### 2.2. Chemistry

**Scheme 1:**
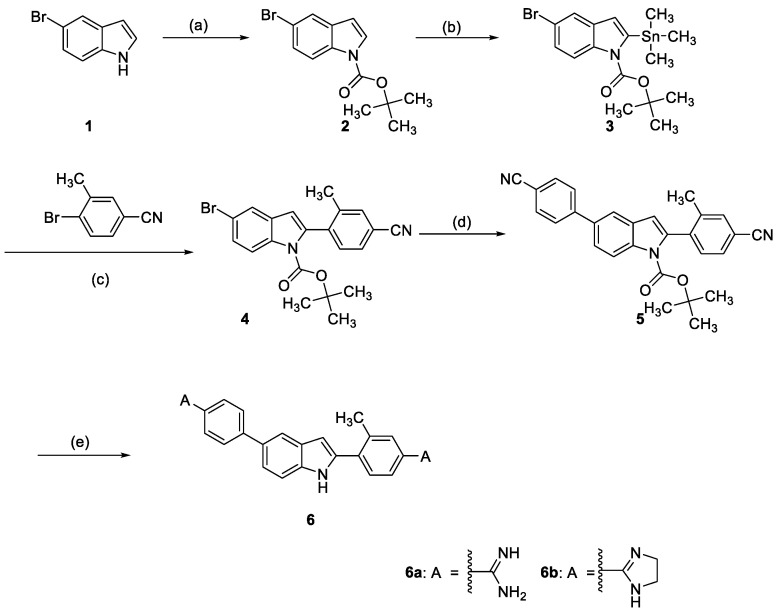
Reagents and conditions: (a) *tert*-Butyldicarbonate, 4-dimethylaminopyridine, CH_2_Cl_2_; (b) Trimethyltin chloride, lithium diisopropylamide/THF; (c) Palladium-tetrakis(triphenylphosphine), dioxane; (d) p-Cyanophenylboronic acid, palladium-tetrakis(triphenylphosphine, sodium carbonate, toluene; (e) For **6a**: i- lithium bis(trimethylsilyl)amide/THF, ii-HCl gas/EtOH; For **6b**: i-HCl gas/EtOH, ii-Ethylene diamine/EtOH.

Scheme 1 shows our approach to synthesize indole-diamidines **6a** and **6b**. Protection of 5-bromoindole **1** was performed using Boc anhydride in presence of DMAP as a catalyst and DCM as a solvent. The Boc-protected derivative **2** was lithiated using LDA in THF at –20 °C followed by in situ conversion into organometalic stannane **3** using trimethyltin chloride [41,42]. Stille coupling of stannane derivative **3** with 4-bromo-3-methylbenzonitrile employing Pd(PPh_3_)_4_ in dioxane [41] afforded compound **4**. Suzuki coupling of bromo derivative **4** [43] with 4-cyanophenylboronic acid using Pd(PPh_3_)_4_ as a catalyst in toluene afforded the dinitrile **5**. The diamidine **6a** was made by reacting the dinitrile **5** with lithium bis(trimethylsilyl)amide [44] in THF then subsequent desilyation using ethanolic HCl. While the imidazoline **6b** was furnished by applying Pinner reaction [45,46] conditions on the dinitrile **5** employing anhydrous ethanolic HCl then reacting the formed intermediate carboximidate with ethylene diamine in ethanol.

**Scheme 2:**
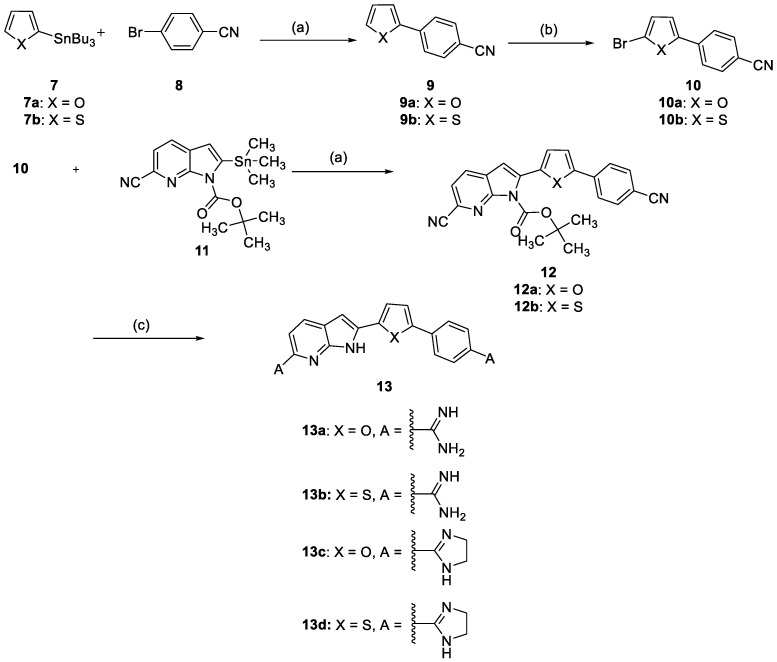
Reagents and conditions; (a) Palladium-tetrakis(triphenylphosphine, dioxane; (b) N-Bromosuccinimide/DMF; (c) For **13a** and **13b**: i- LiN(TMS)_2_/THF, ii- HCl gas/EtOH; For **13c** and **13d**: i- HCl gas/EtOH, ii- Ethylene diamine/EtOH.

The synthesis of the final diamidines **13a-d** is outlined in Scheme 2. Applying Stille cross coupling between the stannane **7a** or **7b** with 4-bromobenzonitrile **8** afforded the cyano compounds **9a** and **9b** in high yield. Compounds **9a** and **9b** were converted to the bromo analogues **10a** and **10b** by bromination using NBS in DMF. The bisnitriles **12a** and **12b** were obtained after coupling of the bromo derivative **10a** and **10b** with the stannane compound **11** utilizing Stille cross coupling conditions. The bisnitriles **12a** and **12b** were converted to the final amidines either by using lithium bis(trimethylsilyl)amide in THF to afford **13a** or **13b** or by applying Pinner reaction condition to afford the imidazolines **13c** or **13d.**

### 2.3. Biological Evaluations

#### 2.3.1. Minimum Inhibitory Concentrations and Spectrum of Activity

Minimum inhibitory concentrations (MICs) and activity spectra were determined utilizing a panel of bacterial isolates and strains consisting of two Gram-positive and two Gram-negative ESKAPE pathogens, in addition to two Gram-positive and three Gram-negative antibiotic-susceptible pathogenic bacteria. The two Gram-positive antibiotic-resistant ESKAPE isolates included the vancomycin-resistant *E. faecium* (VRE; Antibiotic Resistance Isolate Bank #0572) and the methicillin-resistant *S. aureus* (MRSA; strain COL) were used. Additionally, two Gram-positive antibiotic-susceptible bacteria were used including *E. faecalis* (Antibiotic Resistance Isolate Bank # 0671), and methicillin-susceptible *S. aureus* (MSSA ATCC 25923). The utilized Gram-negative antibiotic-resistant ESKAPE clinical isolates included a carbapenem-resistant *Enterobacterale* (CRE) isolate of *K. pneumoniae* (Clinical Isolate #015), and an isolate of carbapenem-resistant *A. baumannii* (CRAB; Clinical Isolate # 03-149.2). Moreover, three Gram-negative antibiotic-susceptible strains were applied namely, *A. baumannii* (ATCC 19606), *P. aeruginosa* (Antibiotic Resistance Isolate Bank # 0238), and *E. coli* (Antibiotic Resistance Isolate Bank # 0017). The results were compared to those of two reference standard drugs including vancomycin against Gram-positive pathogens and gentamicin against Gram-negative bacteria. The results are illustrated in Table 1.

Among the investigated compounds of scaffold type-B, compound **13d,** which structurally consists of a central thiophene ring, a right phenyl ring, and a left pyrrolo[2,3-*b*]pyridine moiety in addition to bis-4,5-dihydro-1*H*-imidazol-2-yl rings as a cyclic amidine moieties, exhibited a remarkable activity. Thus, compound **13d** showed a potential activity against the tested Gram-positive antibiotic-resistant bacterial strains, where it inhibited the growth of both VRE and MRSA with MIC values of 0.25 µg/mL reflecting higher potency than the reference drug, vancomycin, whose MIC values were 8 and 1–2 µg/mL. In addition, compound **13d** inhibited the growth of the two investigated antibiotic-susceptible bacterial strains, *E. faecalis* and MSSA, at MIC values of 1 and 0.5 µg/mL, respectively. These values were also superior to the vancomycin MICs of 2 µg/mL against both strains. As for Gram-negative antibiotic-resistant bacteria, **13d** inhibited the growth of CRE and CRAB at MIC values of 4 and 2 µg/mL, respectively. Again, its activity surpassed the activity of reference standard drug used against Gram-positive bacteria, gentamycin, that showed high MIC values of 64 and >128 µg/mL, respectively. Although compound **13d** showed an MIC value of 0.5 µg/mL that was equal to that of gentamycin against antibiotic-susceptible *E. coli*, compound **13d** showed intermediate to weak activity against the other antibiotic-susceptible Gram-negative bacteria *A. baumannii* and *P. aeruginosa* with MIC values of 32 and >128 µg/mL, respectively. These results suggest that compound **13d** has an excellent activity against *Enterobacterales*, with a variable activity against *Acinetobacter* but poor activity against *Pseudomonas*. It was interesting to find out that compound **13d** elicited a higher activity against antibiotic-resistant bacteria rather than against antibiotic-susceptible bacteria. This might be similar to the known phenomenon of “seesaw effect” in which bacterial resistance mechanisms decrease susceptibility to one drug class but correspondingly increase susceptibility to another drug class [47]. Additionally, it was found that resistance mutations impose complex fitness cost in case of *A. baumannii* [48]. Furthermore, the expression of UPPS was found to have a complex relationship with drug resistance and it is possible that bacteria with resistance to traditional antibacterial agents are more vulnerable to inhibition of UPPS [49]. Moreover, pentamidine analogues were reported as resistance breakers and therefore antibiotic-resistant bacteria might become more susceptible [50]. *P. aeruginosa* is intrinsically resistant to a lot of antibacterials that are active against other types of bacteria due to low permeability, so it is possible that the encountered low activity of compound **13d** against *P. aeruginosa* is because it does not penetrate through its cell in sufficient quantities to kill the bacteria. Interestingly, maintaining all structural features of compound **13d** but replacing the central thiophene ring by the isosteric furan ring afforded compound **13c** that exhibited a marked decrease in the activity. Likewise, compound **13b,** which maintained all structural features of compound **13d** but possessed acyclic unsubstituted amidine moieties in place of the cyclic amidine moieties, exhibited much lower activity relative to compound **13d** against all tested antibiotic-resistant or susceptible Gram-positive as well as Gram-negative bacteria. However, compound **13b** was more active than compound **13c** against antibiotic-susceptible or resistant Gram-negative bacteria. Nevertheless, compound **13b** possessed decreased activity relative to compound **13c** against antibiotic-resistant Gram-positive bacteria and comparable activity against antibiotic-susceptible Gram-positive bacteria. Interestingly, combining both replacement of thiophen and the cyclic amidine by furan and acyclic unsubstituted amidine afforded compound **13a** with partially restored activity against antibiotic-resistant Gram-positive bacteria but not antibiotic-resistant Gram-negative bacteria. Thus, compound **13a** showed moderate activity against VRE and MRSA.

Regarding compounds **6a** and **6b** that possess scaffold type-A that incorporates the larger central indole ring connected to a left phenyl moiety and a right methylphenyl moiety at 5- and 2-positions of the indole ring, respectively, they triggered moderate to good activity against antibiotic-resistant Gram-positive VRE and MRSA. However, both compounds possessed disappointing activity against antibiotic-resistant Gram-negative bacteria. Noteworthy, compound **6a** possessing acyclic unsubstituted amidine moieties was more active relative to compound **6b** having 4,5-dihydro-1*H*-imidazol-2-yl moieties as cyclic amidine fragments. Thus, compound **6a** elicited MIC value of 2 µg/mL against MRSA and MIC value of 32 µg/mL against CRAB. In addition, compound **6a** elicited MIC values of 2 µg/mL against antibiotic-susceptible Gram-positive MSSA as well as 32 µg/mL against Gram-negative CRAB, *A. baumannii*, *P. aeruginosa,* and *E. coli*. Together, these data suggest scaffold distinct structure-activity patterns where the thiophene-based scaffold type-B and 4,5-dihydro-1*H*-imidazol-2-yl moieties as cyclic amidine moieties are the best structural feature combinations to afford an active compound while in indole-based-scaffold type-A the acyclic unsubstituted amidine moieties affords better active compounds.

#### 2.3.2. Time–Kill Assay

**Figure 2:**
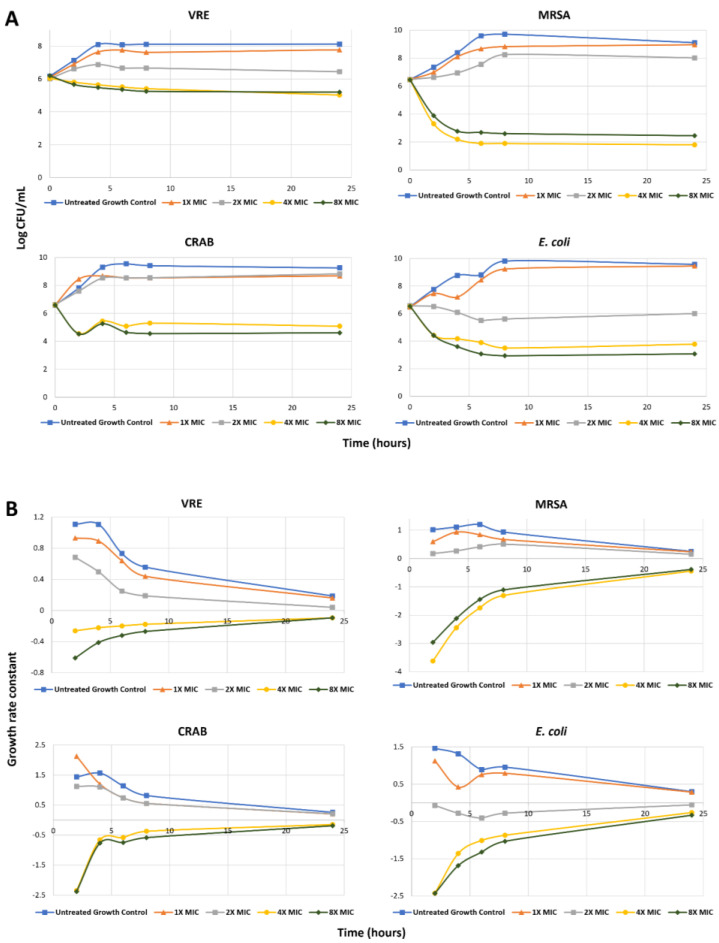
(**A**) Time–kill curve for variable concentrations of compound **13d** against VRE, MRSA, CRAB, and *E. coli* over 24 h; (**B**) Calculated growth rate constants for variable concentrations of **13d** against VRE, MRSA, CRAB, and *E. coli* over 24 h.

As compound **13d** emerged as potential agent against tested antibiotic-resistant Gram-positive and Gram-negative bacteria, it was interesting to explore the pharmacodynamic interactions as a function of both time and concentration [51]. Hence, four different bacterial strains including antibiotic-resistant Gram-positive COL (MRSA) and *E. faecium* AR Bank # 0572 (VRE), in addition to antibiotic-resistant Gram-negative CRAB 03-149.2 (polymyxin-resistant *A. baumannii*) and antibiotic-susceptible Gram-negative *E. coli* AR Bank # 0017, were utilized in 24-h time–killing experiments employing compound **13d** (Figure 2A) and the first order growth rate constants were calculated (Figure 2B). Initially, bacterial growth follows an exponential function characterizing the log phase (rapidly dividing cells phase). After a certain time, the bacterial growth enters a stationary phase (slowly dividing cells phase). In fact, the majority of antibiotics show most of their efficacy during the log-phase of bacterial growth rather than other phases [52]. Accordingly, the action of compound **13d** on different growth phases was assessed. The growth control experiments showed that all of these tested four bacteria were in the initial growth log phase over the first four to six hours, after which the growth rate declined as the bacteria entered the stationary phase. As illustrated in Figure 2, the results indicated that 1X and 2X MIC concentrations of compound **13d** decreased but did not completely stop the growth of VRE, MRSA, and CRAB over the growth log phase. Meanwhile, the 4X and 8X MIC concentrations of compound **13d** not only completely inhibited the growth of VRE, MRSA, and CRAB over the growth log phase, but also achieved sustained killing against all of these antibiotic-resistant bacteria. The calculated first-order growth rate constants for 4X and 8X MIC concentrations were negative over the whole experiment duration indicating that the total number of living bacteria, measured as colony-forming units, remained less than the initial inoculum confirming that the bacteria–kill activity maintained by these concentration of compounds **13d** over log and stationary growth phases. Calculation of the slope of the first-order growth rate showed more negative values over the log-phase rather than the stationary phase, which suggests compound **13d** is more effective in killing VRE, MRSA, and CRAB during the log-phase. Nevertheless, it has a considerable killing effect at the stationary phase as well, indicated by negative first-order growth rate constants over the whole time of each experiment for 4X and 8X MIC concentrations. Interestingly, the measured time–kill dynamics showed that as low as 2X MIC concentration of compound **13d** was effective in killing the antibiotic-susceptible Gram-negative *E. coli*. As the numbers of CFU/mL at almost all sampling times were lower than the initial number of CFU/mL, the calculated growth rate constant of *E. Coli* at 2X MIC concentration showed negative values over almost all follow time (Figure 2B). Together, these results indicate that compound **13d** has potential antibacterial activity against antibiotic-resistant Gram-positive and Gram-negative bacteria as well as antibiotic-susceptible bacteria and might be subject to further development.

### 2.4. Docking Study

As compound **13d** was discovered as a promising agent, it was subjected to docking studies to explore the possible mode of action considering the three molecular targets, viz., UPPS, KARI, and DNA.

#### 2.4.1. Docking Simulation of Compound **13d** to UPPS

**Figure 3:**
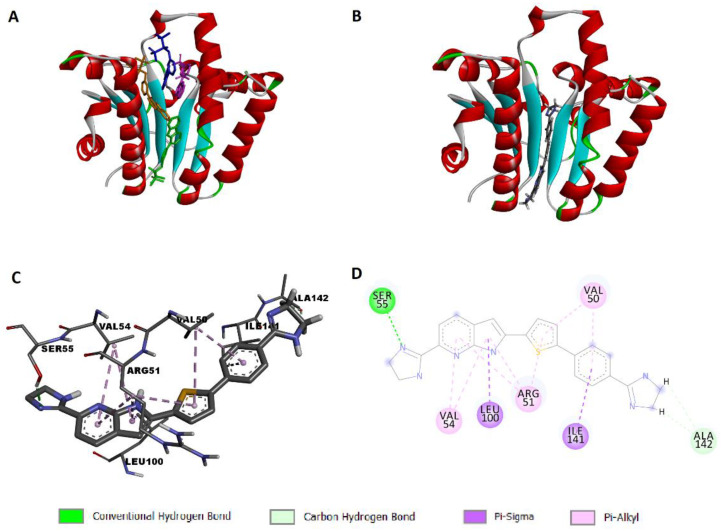
Docking simulation of compound **13d** to 2E98: (**A**) open conformation of the enzyme showing four binding sites with the co-crystallized ligand represented in four different colors in each binding site; (**B**) predicted pose showing compound **13d** docked into site 4 of 2E98; (**C**) predicted 3D pose; (**D**) predicted 2D pose of compound **13d** to 2E98.

Interestingly, the crystal structure of UPPS shows a tunnel-like elongated core, which is enclosed with two α-helices and four β-sheets, with four possible binding sites (Figure 3A). UPPS may adopt two conformations for substrate binding and product release, either opened or closed [53,54]. Accordingly, it was mandatory to carry out docking simulations against both forms of UPPS (PDB IDs: 2E98 and 1X06; respectively) to predict the binding mode, if present, of compound **13d** to UPPS conformer(s) in which active site(s). Results unveiled that our potential antibacterial hit compound could dock successfully to the open conformation of UPPS, specifically, into biding site number four of 2E98 crystal structure as illustrated in Figure 3B. The calculated binding score was −7.9218 Kcal/mol and the predicted binding poses unveiled that the tested compound established a network of favourable binding interactions (Figure 3C,D). The incorporation of two amidine moieties into dihydroimidazole rings was proved to cause a remarkable increase in the potency of compound **13d** and this might be explained by docking simulation where the predicted binding mode unveiled that one of the moieties formed hydrogen bond with Ser55 amino acid residue in the binding site of 2E98 and the other formed carbon hydrogen interaction with Ala142. In addition, the central thiophene ring formed two π-alkyl interactions with Val50 and Arg51. The right phenyl ring established favourable π-sigma interaction with Ile141 and π-alkyl interaction with Val50 while the left pyrrolo[2,3-*b*]pyridine formed various π-alkyl and π-sigma interactions with Arg51, Val54, and Leu100. Collectively, it is anticipated that compound **13d** docked into the open conformation of UPPS specifically in binding site 4 (Figure 3).

#### 2.4.2. Docking Simulation of Compound **13d** to KARI

**Figure 4:**
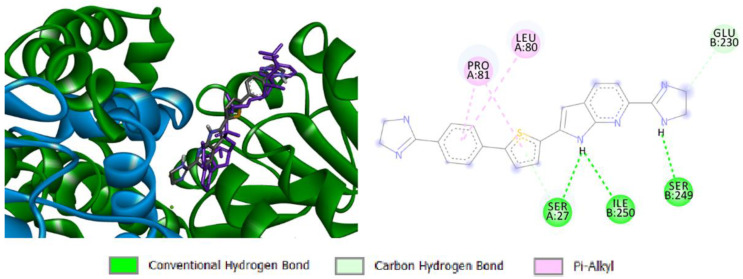
Docking simulation of compound **13d** to dimeric 7KH7: 3D representation showing the first monomeric chain in green color, the intertwined C-domain of the other chain in blue color, and NADPH in violet color (**left**) and bonding interactions of the docked compound (**right**).

It is reported that there are two classes of KARI enzymes namely, class I and II. They differ in the length of polypeptide chain, where class I has about 340 amino acid residues while class II has extra polypeptide segment. Moreover, class I enzymes are usually found within microorganisms while those belonging to class II are typically found in plants. Enzymes from both classes are composed of a dimeric structure with two intertwined C-domains with the active site is located between these two domains [55]. Activity of KARI is essentially dependent on two Mg^2+^ ions and NADPH cofactor. Interestingly, the location of these ions is highly dependent upon whether the enzyme is NADPH-bound or not. This suggested that the two ions have non-significant role in the catalysis process of KARI. Moreover, it was observed that binding of KARI and its cofactors aids widening the active site for substrate binding. Consequently, KARI inhibitors might bind to either NADPH-bound or unbound forms of the enzyme [56]. Accordingly, a docking experiment was carried out in the presence and absence of NADPH to explore whether compound **13d** would dock into the bound or unbound enzyme. Calculations unveiled that compound **13d** docked successfully into the NADPH-unbound form of the enzyme with respectable binding score of −7.5505 Kcal/mol. The predicted 3D-pose of the docking simulation clarified that the dihydroimidazole moiety attached to the left pyrrolo[2,3-*b*]pyridine ring is located near the interwind C-domain of the second monomeric chain (Figure 4). Additionally, this side of the ligand was found to form favorable hydrogen bonds between the NH of the dihydroimidazole moiety and Ser249 and another hydrogen bond between NH of the pyrrolo[2,3-*b*]pyridine and Ile250. Additionally, the first monomeric chain was involved in a network of favorable binding interactions with the docked molecule including carbon hydrogen and π-alkyl interactions between the central thiophene and the phenyl rings with Ser27, Leu80, and Pro81. These results ascertained that compound **13d** acts as KARI inhibitor via binding to the NADPH-unbound form of the enzyme.

#### 2.4.3. Docking Simulation of Compound **13d** to Bacterial DNA

**Figure 5:**
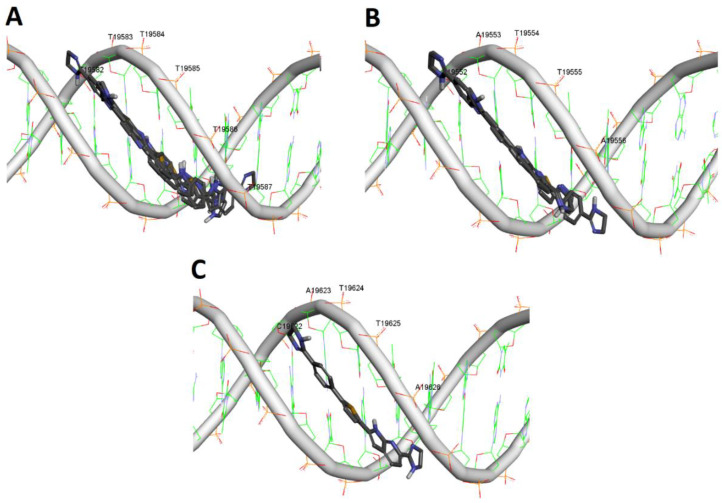
Docking simulation of compound **13d** to DNA sequence of *S. aureus* (19,540–19,644): (**A**) Anticipated poses of compound **13d** to TTTTTT sequence 19,582–19,587; (**B**) Anticipated poses of compound **13d** to TATTA sequence 19,552–19,556; (**C**) Anticipated poses of compound **13d** to CATTA sequence 19,622–19,626.

Docking simulation of compound **13d** to DNA of *S. aureus* was performed to explore the potential selective binding to bacterial DNA. Accordingly, a 105-mer sequence of *S. aureus* TY4, ETB plasmid DNA was obtained from GenBank (accession No. AP003088, sequence 19,540–19,644). A preliminary docking study was conducted to broadly identify the possible sites for binding, followed by another more accurate run utilizing the identified possible sequences in the first experiment. It was found that compound **13d** docked into the minor groove of B-DNA, not the A-conformation, with calculated binding scores ranging from −9.2059 to −9.0894 Kcal/mol for the top ten poses of the docking experiment. In fact, six of the top ten poses within the docking simulation were bound to the sequence TTTTTT 19,582–19,587, three poses were predicted to bind with sequence TAATTA 19,553–19,556, and only one pose was bound to sequence CATTA 19,622–19,626 as illustrated in Figure 5. These results suggested that compound **13d** is a potential MGB that possibly binds mainly to sequence TTTTTT 19,582–19,587.

## 3. Materials and Methods

### 3.1. Chemistry

Synthesis and compounds characterization are as detailed in the supplementary materials.

### 3.2. Biological Evaluations

Biological evaluations were performed following standard protocols [6,57] as detailed in the supplementary materials.

### 3.3. Docking Simulations

The docking simulations were carried out adopting reported procedures as detailed in the supplementary materials.

## 4. Conclusions

Towards the discovery of novel antibacterial agents against antibiotic-resistant pathogens, novel hybrid molecules of two scaffold types were designed, synthesized, and evaluated. Compound **13d,** which possesses a type-B scaffold, was the most promising compound and elicited potential activity against Gram-positive and Gram-negative bacteria with various resistance profiles including VRE, MRSA, CRE, CRAB, MSSA, *E. faecalis*, *E. coli*, *A. baumannii*, and *P. aeruginosa*. Study of time–kill activity of compound **13d** showed that it achieved sustained killing against tested bacteria at 4X MIC concentrations with maximum killing observed during the log phase of bacterial growth. *In silico* simulations suggested compound **13d** is a multifunctional molecule that possibly interacts with bacterial UPPS, KARI, and DNA as potential targets. Overall, compound **13d** represents a promising hit for future development of potential antibacterial agents against antibiotic-resistant as well as antibiotic-susceptible bacteria.

## Figures and Tables

**Table 1:** Determined MIC (μg/mL) for the investigated compounds and reference drugs against antibiotic-resistant Gram-positive and Gram-negative ESKAPE pathogens and antibiotic-susceptible Gram-positive and Gram-negative bacteria.

Compound	MIC (μg/mL)								
	Antibiotic-Resistant Gram-Positive		Antibiotic-Susceptible Gram-Positive		Antibiotic-Resistant Gram-Negative		Antibiotic-Susceptible Gram-Negative		
	*E. faecium* (VRE) ^a^	*S. aureus* (MRSA) ^b^	*E. faecalis*	*S. aureus* (MSSA) ^c^	*K. pneumonia* (CRE) ^d^	*A. baumannii* (CRAB) ^e^	*A. baumannii*	*P. aeruginosa*	*E. coli*
**6a**	16	2	>128	2	>128	32	32	32	32
**6b**	32	32	>128	32	>128	128	128	>128	128
**13a**	16	8	>128	32	>128	128	32	16	32
**13b**	64	32	>128	32	>128	64	64	64	32
**13c**	32	32	>128	32	>128	128	128	>128	32
**13d**	0.25	0.25	1	0.5	4	2	32	>128	0.5
Vancomycin	8	1–2	2	2	-	-	-	-	-
Gentamycin	-	-	-	-	64	>128	8	2	0.5

^a^ VRE: Vancomycin-resistant *Enterococcus faecium*; ^b^ MRSA: Methicillin-resistant *Staphylococcus aureus*; ^c^ MSSA: Methicillin-susceptible *Staphylococcus aureus*; ^d^ CRE: Carbapenem-resistant *Enterobacterale* (*Klebsiella pneumonia*); ^e^ CRAB: Carbapenem-resistant *Acinetobacter baumannii.*

## Data Availability

Data is contained within the article or supplementary material.

## Supplementary Materials

The following supporting information can be downloaded at: https://www.mdpi.com/article/10.3390/ph16071040/s1, Chemical synthesis procedures and characterization data; Biological evaluations protocols; *In silico* calculations methods.
