# Supplementary material for: Small Molecules Incorporating Privileged Amidine Moiety as Potential Hits Combating Antibiotic-Resistant Bacteria
Source: Pharmaceuticals (Basel). 2023 Jul 22;16(7):1040. doi: 10.3390/ph16071040 (PMC10384254; doi:10.3390/ph16071040)
Supplement: Supplementary file 1 [file pharmaceuticals-16-01040-s001.zip › pharmaceuticals-2457220-supplementary.pdf]

Supplementary Information for

Small molecules incorporating privileged amidine moiety as potential hits combating antibiotic-resistant bacteria

Selwan M. El-Sayed [1,2,‡], Samar A. Ahmed [3,‡], Kanika Gulia [4], Justin R. Lenhard [3], Ahmed H.E. Hassan [1,5,*], and Abdelbasset A. Farahat [4,6,*]

[1] Department of Medicinal Chemistry, Faculty of Pharmacy, Mansoura University, Mansoura 35516, Egypt; salwanmahmoud@mans.edu.eg (S.M.E-S.); ahmed_hassan@mans.edu.eg (A.H.E.H.)

[2] Pharmaceutical Chemistry Department, Faculty of Pharmacy, Delta University for Science and Technology, Gamasa 35712, Egypt

[3] Department of Clinical and Administrative Sciences, College of Pharmacy, California Northstate University, Elk Grove, CA 95757, USA; samar.ahmed@cnsuedu.onmicrosoft.com (S.A.A.); justin.lenhard@cnsu.edu (J.R.L.)

[4] Master of Pharmaceutical Sciences Program, California Northstate University, 9700 W Taron Dr., Elk Grove, CA 95757, USA; abdelbasset.farahat@cnsu.edu (A.A.F.); kanika.gulia7488@cnsu.edu (K.G.)

[5] Medicinal chemistry laboratory, College of Pharmacy, Kyung Hee University, Seoul 02447, Republic of Korea;

[6] Department of Pharmaceutical Organic Chemistry, Faculty of Pharmacy, Mansoura University, Mansoura 35516, Egypt

‡ Both of Selwan M. El-Sayed and Samar A. Ahmed contributed equally and should be recognized as first authors

***Corresponding authors:**

* Correspondence: abdelbasset.farahat@cnsu.edu (A.A.F.) and ahmed_hassan@mans.edu.eg (A.H.E.H.)

1. Chemistry

All commercial reagents were used without purification. Melting points were determined on a Mel-Temp 3.0 melting point apparatus and are uncorrected. TLC analysis was carried out on silica gel 60 F254 precoated aluminum sheets using UV light for detection. ^{1}H and ^{13}C NMR spectra were recorded on a Bruker 600 MHz spectrometer (except as noted) using the indicated solvents. Mass spectra were obtained from the Georgia State University Mass Spectrometry Laboratory, Atlanta, GA. Elemental analysis were performed by Atlantic Microlab Inc., Norcross, GA. Compound 5 was made according to the published procedure [1]. Compound 11 was made according to the published procedure [2]. Compounds 13c and 13d were prepared by the same method used to prepare compound 6b.

4-(5-(4-Carbamimidoylphenyl)-1*H*-indol-2-yl)-3-methylbenzimidamide (6a).

The dinitrile **5** (0.66 mmol) was suspended in freshly distilled THF (5 mL) and treated with lithium trimethylsilyl amide 1M solution in tetrahydrofuran (4 mL, 3.98 mmol), the mixture was stirred for 2 days at room temperature. The reaction mixture was then cooled to zero °C and HCl saturated ethanol (2 mL) was added. The mixture was stirred for 24 h, diluted with ether and the resultant solid was collected by filtration. The diamidine was purified by neutralization with 1*N* sodium hydroxide solution followed by filtration of the formed solid and washing with water and dried. Finally, the free base was stirred with ethanolic HCl for one week to make sure that the $(Boc)_2O$ group was completely removed, diluted with ether, and the solid formed was filtered and dried to give the final diamidine.

Yellow solid, yield 72 %. mp > 300 °C ; ^{1}HNMR (DMSO-d_6, 600 MHz) δ 11.90 (s, 1H), 9.51 (s, 2H), 9.47 (s, 2H), 9.31 (m, 2H), 9.26 (m, 2H), 8.03 (brs, 1H), 7.96 (m, 4H), 7.91 (brs, 1H), 7.85 (m, 2H), 7.59 (brs, 2H), 6.87 (d, 1H, J = 1.8 Hz), 2.61 (s, 1H); ^{13}CNMR (DMSO-d_6, 150 MHz) δ

165.8, 165.6, 147.3, 137.6, 137.2, 136.6, 131.3, 130.4, 129.5, 129.4, 129.3, 129.2, 127.2, 126.9, 126.2, 125.8, 122, 119.6, 112.7, 104.7, 21.9; HR-MS: m/z calc. for $C_{23}H_{22}N_5$: 368.1875, found 320.1884 $[M+1]^+$; Anal. Calcd. For $C_{23}H_{21}N_5$. 2HCl. $0.5H_2O$: C, 61.47; H, 5.38; N, 15.58. Found: C, 61.39; H, 5.36; N, 15.22.

2-(4-(4,5-Dihydro-1*H*-imidazol-2-yl)-2-methylphenyl)-5-(4-(4,5-dihydro-1*H*-imidazol-2-yl)phenyl)-1*H*-indole (6b).

The dinitrile **5** (2.1 mmol) were suspended in saturated ethanolic-HCl (gas) and stirred at room temperature for 1 week, with complete isolation from air. Anhydrous ether was added and the crystals which formed were filtered, dried under vacuum for 30 min and then suspended in anhydrous ethanol. Ethylene diamine (4.5 mmol) was added to the solution and the solution was stirred at room temperature for 2 days and then refluxed for 2 days and left to cool down. Anhydrous ether was added, and the precipitated crystals (HCl salt) were filtered. The final compounds were purified by neutralization with 1N sodium hydroxide solution followed by filtration of the formed free base that was washed with water and dried. The free base was stirred with ethanolic-HCl (gas) for 2 days, diluted with anhydrous ether, and the solid which formed was filtered and dried to give the final diamidine.

Yellow solid, yield 39 %. mp > 300 °C ; ^{1}HNMR (DMSO-d_6, 600 MHz) δ 11.95 (s, 1H), 10.96 (s, 2H), 10.86 (s, 2H), 8.17 (m, 3H), 8.07 (m, 2H), 7.99 (d, 2H, J = 9 Hz), 7.90 (d, 1H, J = 7.8 Hz), 7.63 (d, 1H, J = 9 Hz), 7.59 (d, 1H, J = 9 Hz), 6.90 (brs, 1H), 4.01 (brs, 8H), 2.61 (s, 1H); ^{13}CNMR (DMSO-d_6, 150 MHz) δ 164.9, 164.7, 147.8, 138, 137.7, 137, 136.7, 132, 130.2, 129.8, 129.6, 129.3, 127.4, 126.8, 122.2, 121, 120.1, 119.8, 112.7, 105, 44.8, 44.7, 21.9; HR-MS: m/z calc. for $C_{27}H_{26}N_5$ 420.2188, found 420.2183 $[M+1]^+$; Anal. Calcd. For $C_{27}H_{25}N_5$. 2HCl. H_2O: C, 63.52; H, 5.72; N, 13.71. Found: C, 63.59; H, 5.61; N, 13.49.

General method for the preparation of the bis-nitriles 12a,b.

Tetrakistriphenylphosphine palladium (0.288 g, 0.25 mmol) was added to a stirred mixture of *tert*-butyl 6-cyano-2-(trimethylstannyl)-1*H*-pyrrolo[2,3-*b*]pyridine-1-carboxylate, (2.03 g, 5 mmol) and the appropriate bromo derivative **10** (5 mmol) in deaerated dioxane (20 mL) under a nitrogen atmosphere. The vigorously stirred mixture was warmed to 90–100 °C for 24 h. The solvent was evaporated under reduced pressure, the formed residue was partitioned between ethyl acetate (100 mL) containing 5 mL ammonia to remove the palladium species, then washed with water, passed through celite to remove the catalyst, dried over magnesium sulphate, and vacuum-dried. Purification was done using column chromatography on silica gel, and hexanes/ethyl acetate (80/20, v/v) as an eluent.

***tert*-Butyl 6-Cyano-2-(5-(4-cyanophenyl)furan-2-yl)-1*H*-pyrrolo[2,3-*b*]pyridine-1-carboxylate (12a).**

White solid, yield 69 %. mp 201-202 °C ; ^{1}HNMR (DMSO-d_6, 400 MHz) δ 9.21 (d, 1H, *J* = 2Hz), 8.45 (brs, 1H), 8.93 (dd, 1H, *J* = 8Hz, 2Hz), 8.13 (d, 1H, *J* = 8Hz), 7.9 (d, 1H, *J* = 8Hz), 7.72 (d, 1H, *J* = 8Hz), 7.6 (d, 1H, *J* = 3.6Hz), 7.28 (s, 1H), 7.12 (d, 1H, *J* = 3.6Hz), 1.42 (s, 9H); ^{13}CNMR (DMSO-d_6, 100 MHz) δ 149.7, 148.1, 146.3, 136.4, 132.1, 131.7, 130.6, 128.9, 128.6, 126.4, 121.8, 120.2, 119.8, 117.3, 113.5, 111.7, 111, 108.2, 85.5, 27.5; ESI-MS: m/z calculated for $C_{24}H_{18}N_4O_3$: 410.42, found: 411.1 $[M+1]^+$; Anal. Calcd. For $C_{24}H_{18}N_4O_3$: C, 70.23; H, 4.42; N, 13.65. Found: C, 69.90; H, 4.38; N, 13.40.

***tert*-Butyl 6-cyano-2-(5-(4-cyanophenyl)thiophen-2-yl)-1*H*-pyrrolo[2,3-*b*]pyridine-1-carboxylate (12b).**

Brown solid, yield 62 %. mp 208-210 °C; ^{1}HNMR ($CDCl_3$, 400 MHz) δ 8.84(d, 1H, *J* = 1.6Hz), 8.55 (brs, 1H), 7.99 (dd, 1H, *J* = 8.4Hz, 2Hz), 7.78 (d, 1H, *J* = 8Hz), 7.7 (d, 1H, *J* = 3.6Hz), 7.67

(d, 1H, J = 8Hz), 7.55 (dd, 1H, J = 8Hz, 1.6Hz), 7.24 (d, 1H, J = 3.6Hz), 6.83 (s, 1H), 1.49 (s, 9H); ^{13}CNMR ($CDCl_3$, 100 MHz) δ 149.6, 148.1, 148, 146.3, 136.4, 132, 131.7, 130.7, 128.8, 128.6, 126.3, 121.4, 120.2, 119.8, 117.2, 113.4, 111.7, 111, 108.2, 85.4, 27.9 ; ESI-MS: m/z calculated for $C_{24}H_{18}N_4O_2S$: 426.49, found: 427.5 $[M+1]^+$; Anal. Calcd. For $C_{24}H_{18}N_4O_2S$: C, 67.59; H, 4.25; N, 13.14. Found: C, 67.84; H, 4.33; N, 12.99.

2-(5-(4-Carbamimidoylphenyl)furan-2-yl)-1*H*-pyrrolo[2,3-*b*]pyridine-6-carboximidamide (13a).

Compound **13a** was prepared by the same method used to prepare compound **6a**.

Yellow solid, yield 55 %. mp > 300 °C ; ^{1}HNMR (DMSO-d_6, 600 MHz) δ 9.56 (brs, 3H), 9.46 (s, 2H), 9.36 (s, 2H), 9.34 (s, 2H), 8.23 (d, 1H, J = 8.4Hz), 8.14 (d, 2H, J = 8.4Hz), 8.02 (m, 4H), 7.48 (d, 1H, J = 3.6Hz), 7.50 (d, 1H, J = 3.6Hz), 7.12 (d, 1H, J = 1.8Hz); ^{13}CNMR (DMSO-d_6, 150 MHz) δ 165.3, 163.6, 152.8, 148.7, 147.3, 137.1, 134.6, 134.2, 129.4, 129.1, 126.9, 125.2, 124.3, 116.2, 112.7, 112, 98.1; HR-MS: m/z calc. for $C_{19}H_{17}N_6O$ 345.1464, found 345.1478 $[M+1]^+$; Anal. Calcd. For $C_{19}H_{16}N_6O$. 2HCl. $0.5H_2O$: C, 53.53; H, 4.49; N, 19.71. Found: C, 53.48; H, 4.22; N, 19.39.

2-(5-(4-Carbamimidoylphenyl)thiophen-2-yl)-1*H*-pyrrolo[2,3-*b*]pyridine-6-carboximidamide (13b).

Compound **13b** was prepared by the same method used to prepare compound **6a**.

Yellow solid, yield 77 %. mp > 300 °C; ^{1}HNMR (DMSO-d_6, 400 MHz) δ 12.73 (brs, 1H), 9.74 (s, 2H), 9.44 (s, 2H), 9.39 (s, 2H), 9.14 (s, 2H), 9.02 (s, 1H), 8.34 (dd, 1H, J = 8.4Hz, 1.6Hz), 8.22 (d, 1H, J = 8.4Hz), 8.10 (d, 1H, J = 3.6Hz), 7.98 (brs, 1H), 7.73 (d, 1H, J = 3.6Hz), 7.47 (dd, 1H, J = 8.4Hz, 1.6Hz), 6.99 (d, 1H, J = 1.6Hz); ^{13}C NMR (DMSO-d_6, 100 MHz) δ 166.5, 163.5, 155.3, 149.2, 142.6, 138.3, 137.2, 136.2, 136.0, 132.5, 129.0, 126.9, 122.1, 120.8, 120.4, 119.1, 118.1,

112.1, 100.4; HR-MS: m/z calc. for $C_{19}H_{17}N_6S$ 361.1235, found 361.1250 $[M+1]^+$; Anal. Calcd. For $C_{19}H_{16}N_6S$. 2HCl. 0.5H_2O: C, 51.58; H, 4.32; N, 18.99. Found: C, 51.33; H, 4.11; N, 18.67.

2. Biological evaluations

2.1. Evaluation of spectrum and minimum inhibitory concentrations (MICs) against ESKAPE pathogens

The MICs of the investigated compounds were determined by applying the broth microdilution assay per specifications from the Clinical and Laboratory Standards Institute [3]. In brief, 96-well trays with a maximum volume of 0.5 ml per well were used for susceptibility testing. On the day of each experiment, an overnight culture of bacteria was used to create a turbidity adjusted concentration of organism suspended in calcium and magnesium adjusted Mueller-Hinton broth. Antibacterial stock solutions purchased from AK Scientific were created on the day of each experiment, and a series of two-fold dilutions were used to create a concentration gradient in rows of the tray. The initial inoculum of bacteria in each well was ~ 5 x 10^5 CFU/mL suspended in 150 µL of total volume. Completed trays were covered in tape to prevent desiccation, and visible growth was recorded after 18 – 24 hours of incubation at 37°C. Study compounds were evaluated along with vancomycin or gentamicin to ensure consistency with other laboratories. All experiments were completed in quadruplicate. If the results of four experiments were evenly split between two MIC values, then the MIC of the compound was reported as a range between the two values.

2.2. Time-kill assay

Time-kill assay was performed adopting the reported protocol [4]. Four different bacterial strains including *E. faecium* AR Bank # 0572 (VRE), COL (MRSA), 03-149.2 (polymyxin-resistant *A.*

baumannii), and *E. coli* AR Bank # 0017 were utilized. Overnight cultures of bacteria and cation-adjusted Mueller-Hinton broth was used to attain 20 mL suspensions with starting inocula of ~10^6 CFU/mL, which were incubated at 37°C in a water bath with constant shaking. Concentration arrays of compound 13d were prepared for each organism corresponding to 1X, 2X, 4X and 8X the MIC of the compound. At 0, 2, 4, 6, 8 and 24 hours, 100 µL samples were collected from each reaction vessel, serially diluted in saline, and plated onto Mueller–Hinton agar for viable cell counting. After 24 hours of incubation at 37°C, colonies were enumerated to quantity the bacteria in each experiment.

3. Docking simulations

Regarding UPPS docking study, its crystal structure was obtained from Protein Data Bank (PDB ID: 1X06 and 2E98 for closed and opened conformations, respectively). Structure preparation was performed regarding each target as well as ligand, docking was performed applying the co-crystallized ligands' binding sites. Obtained results were examined carefully, analyzed, and visualized using Discovery Studios Visualizer. Similarly, KARI's crystal structure was downloaded from PDB (ID: 7KH7) and docking experiment was performed once in presence of NADPH and other in absence of it. The docking study against *S. aureus* DNA was performed utilizing a 105-mer sequence TY4, ETB plasmid DNA (GenBank accession No. AP003088, sequence 19540-19644) was downloaded from GenBank. Two experiments were conducted, the first was preliminary in order to get possible sites over the 105 sequence and the other was performed on the identified sequences in the first study.

NMR Spectra

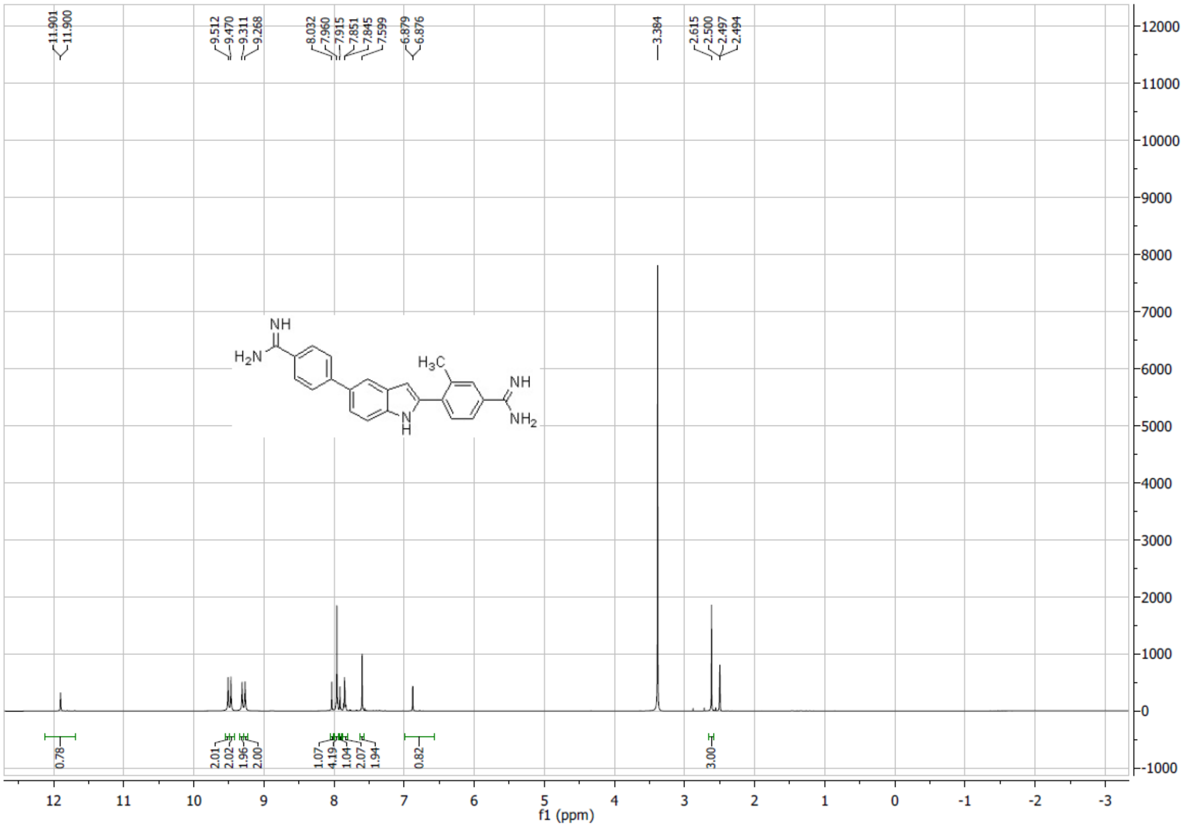

^{1}H NMR Spectra of compound 6a

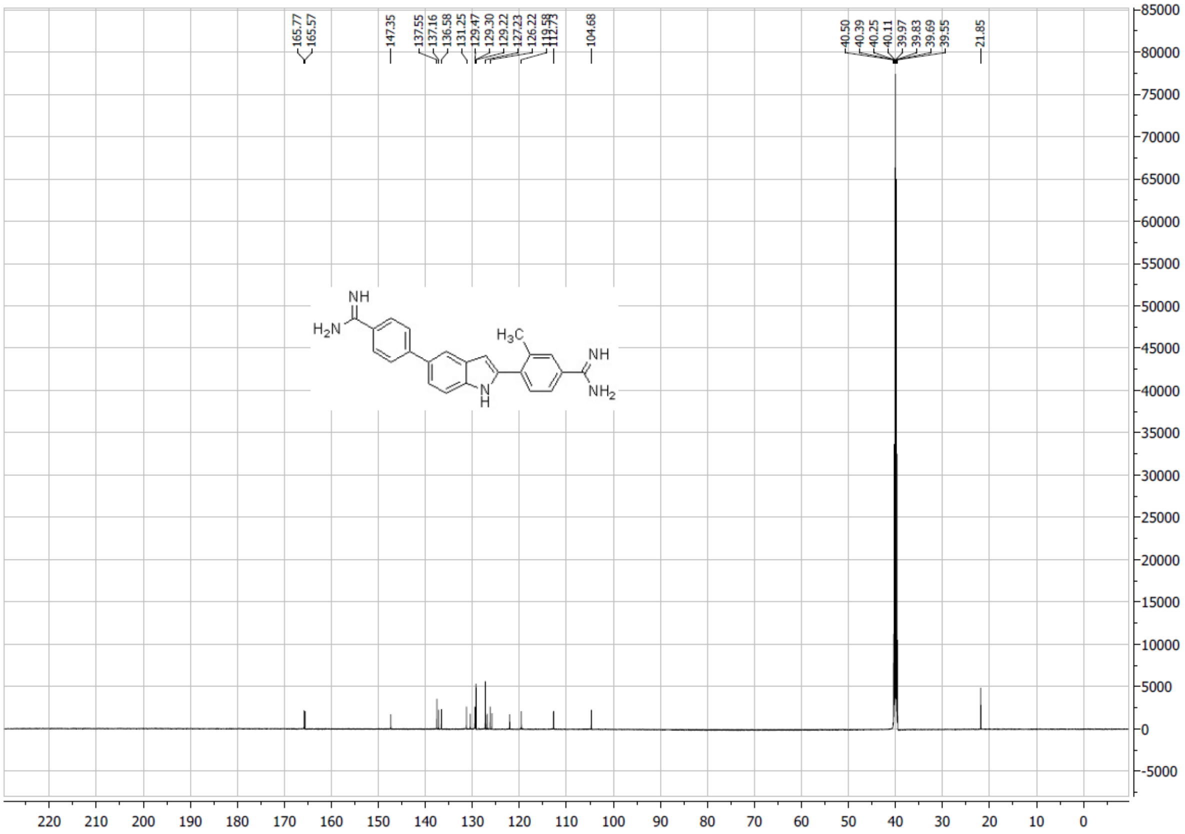

^{13}C NMR Spectra of compound 6a

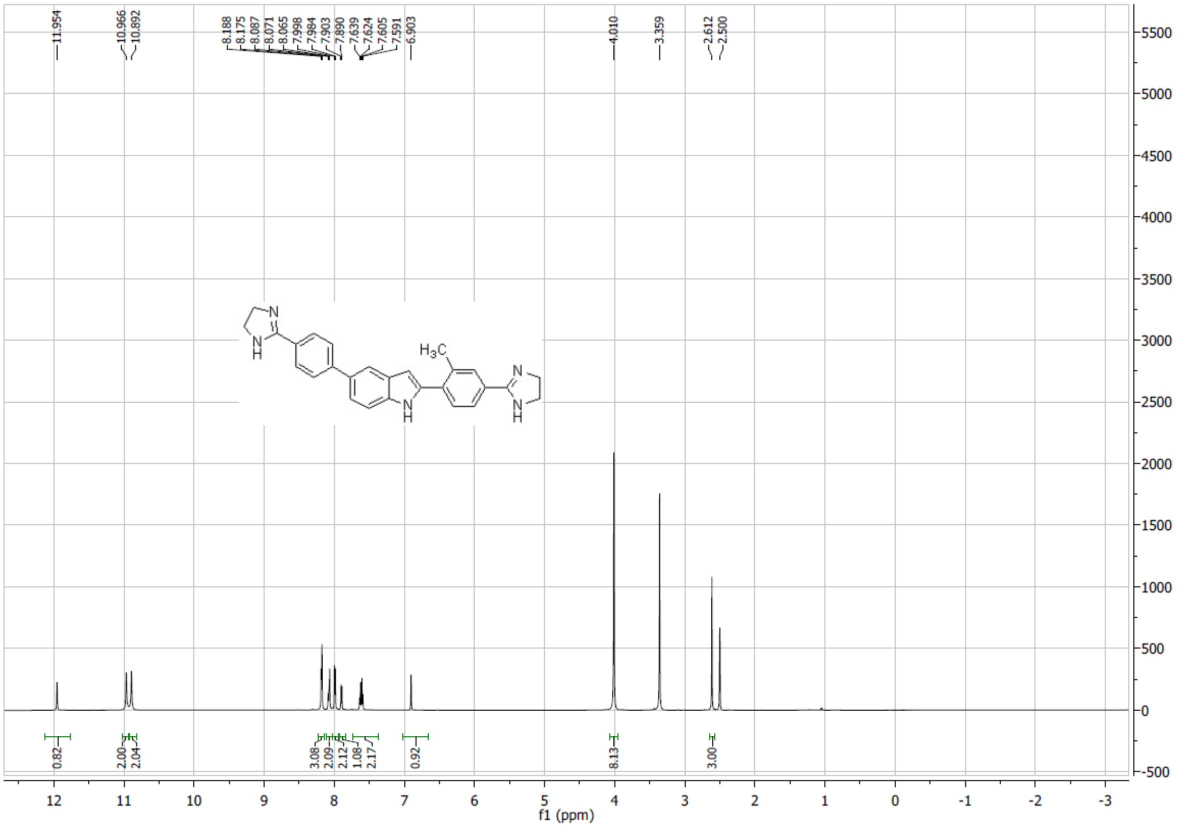

^{1}H NMR Spectra of compound 6b

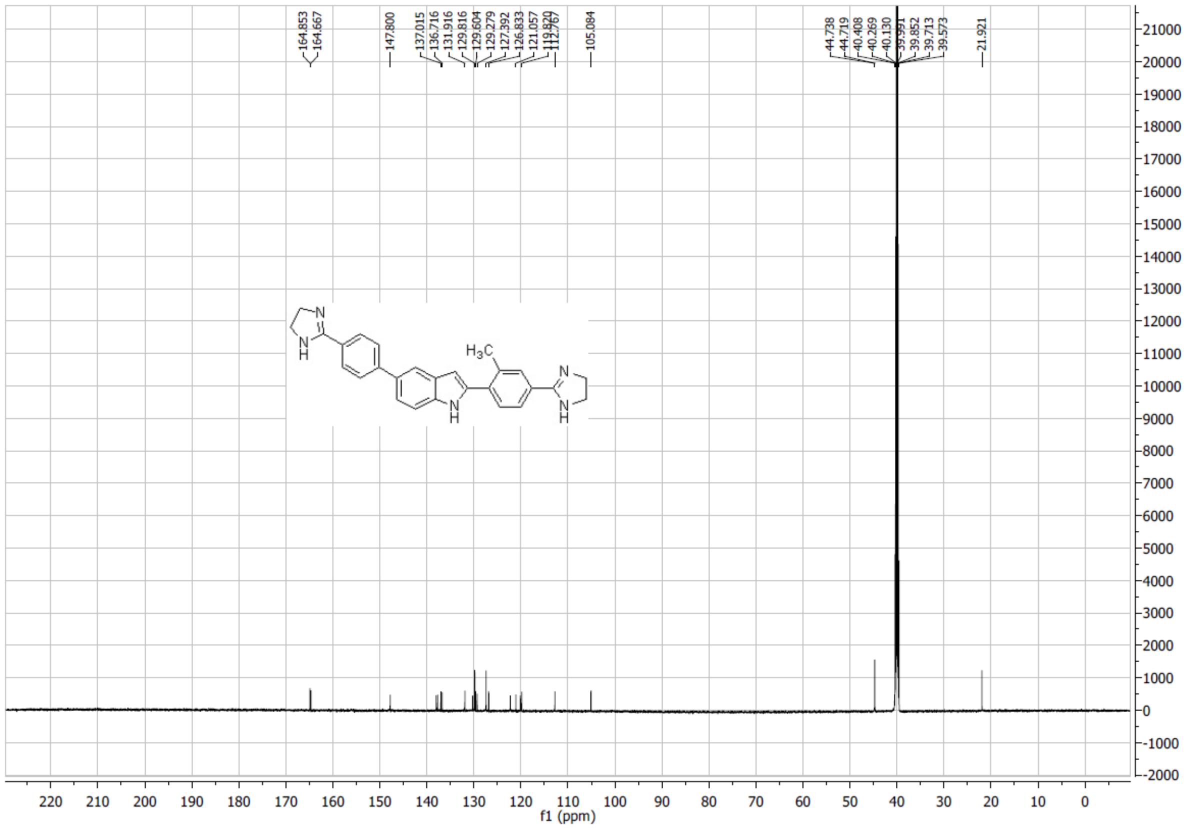

^{13}C NMR Spectra of compound 6b

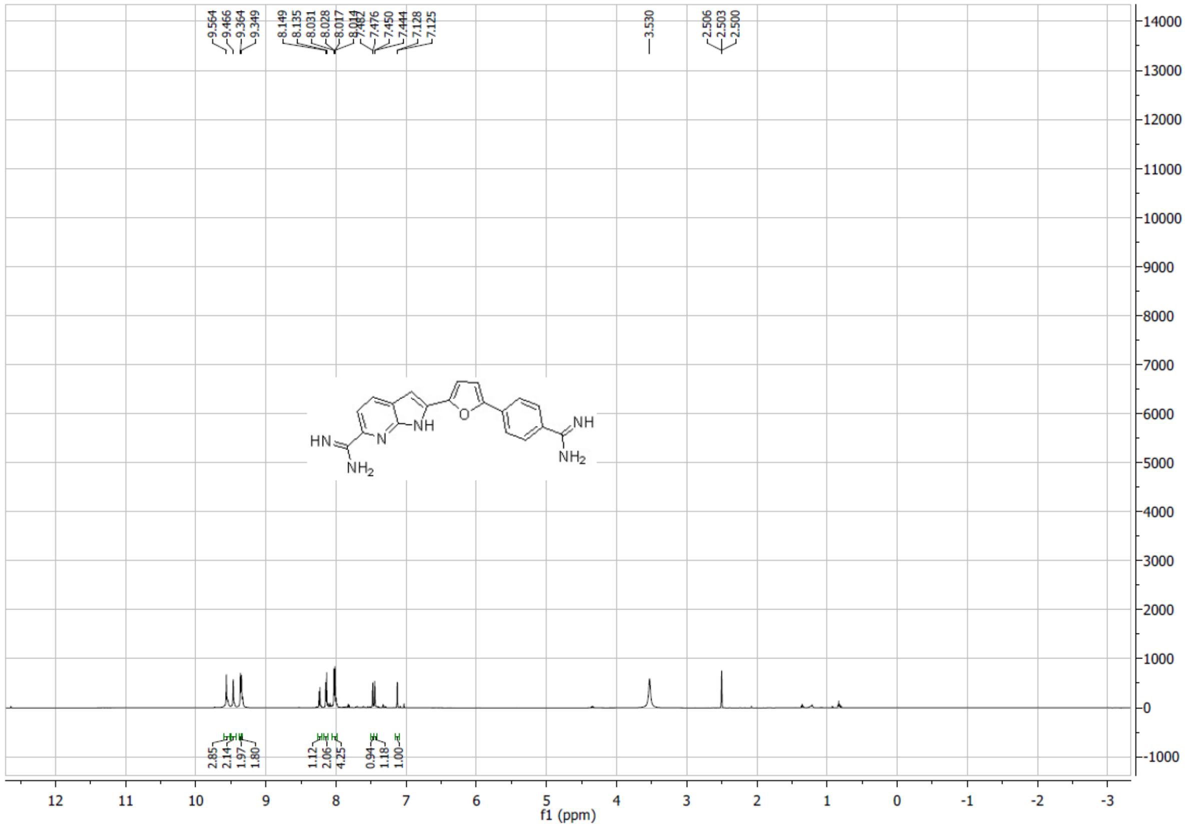

^{1}H NMR Spectra of compound 13a

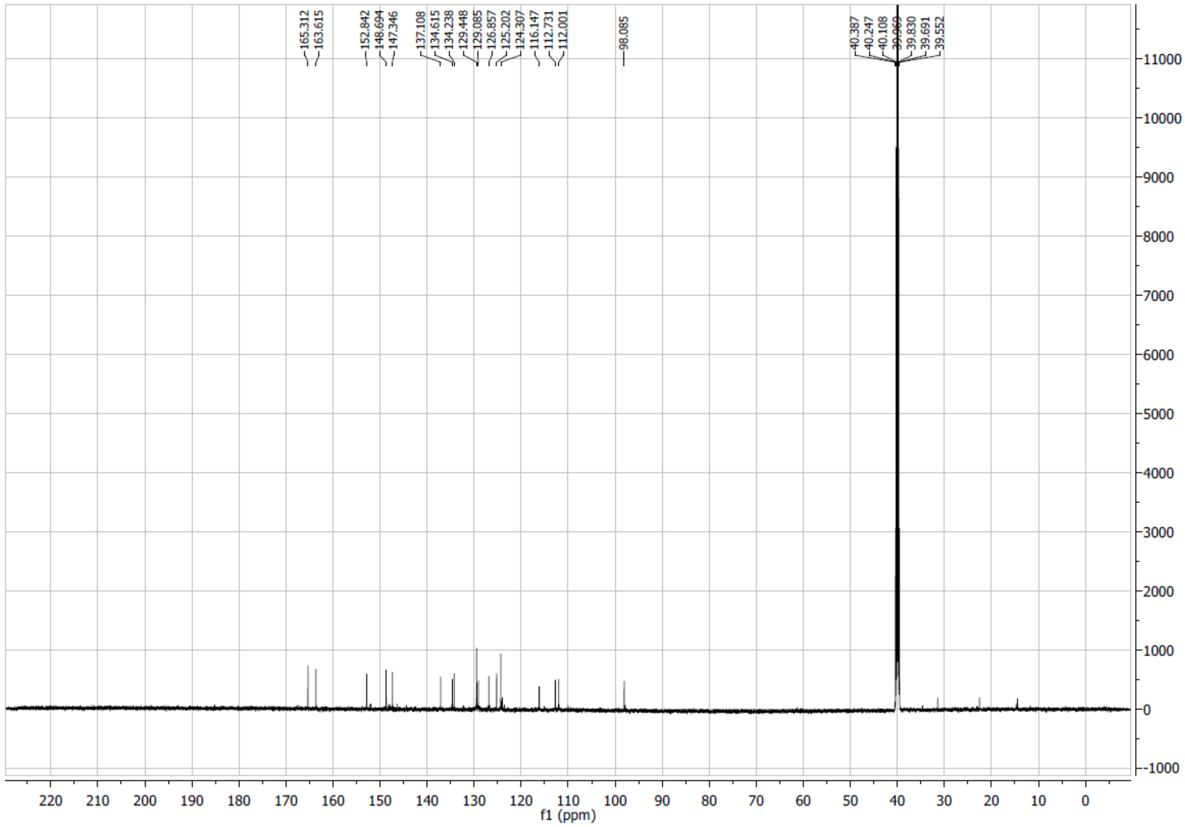

^{13}C NMR Spectra of compound 13a

High Resolution Mass Spectra

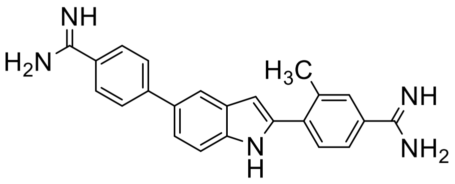

6a

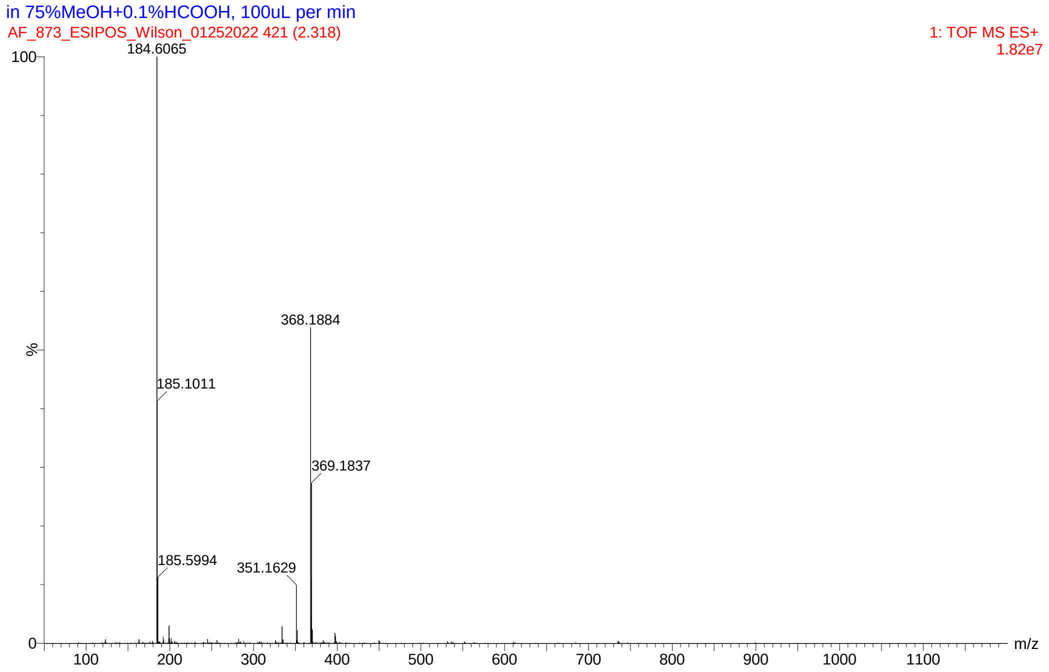

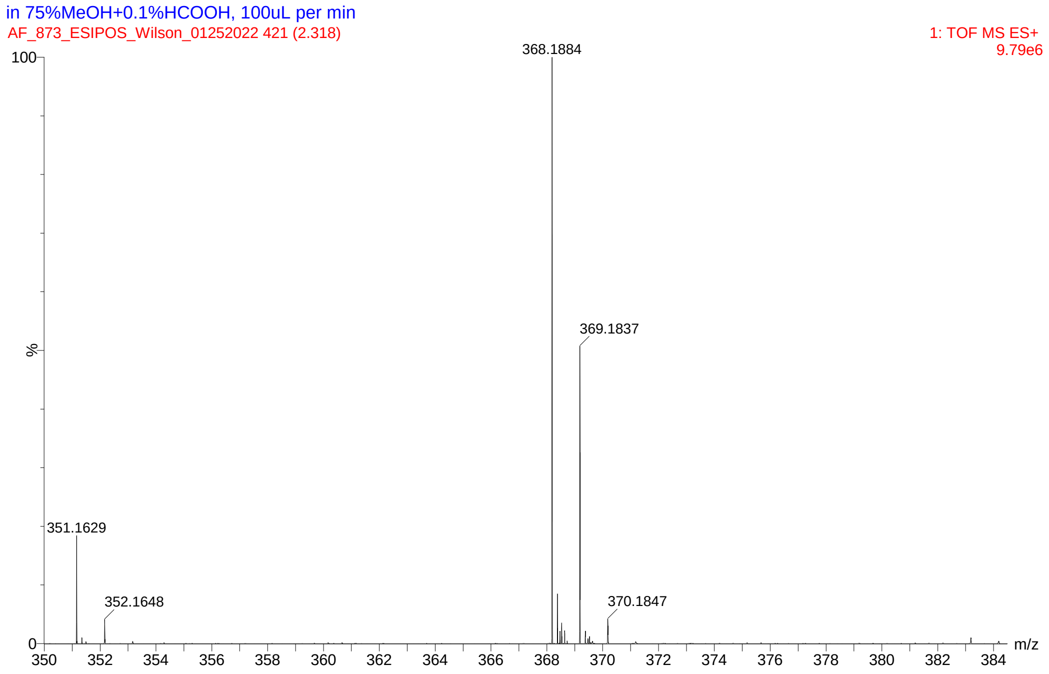

Elemental Composition Report

Single Mass Analysis

Tolerance = 5.0 PPM / DBE: min = -1.5, max = 50.0

Element prediction: Off

Monoisotopic Mass, Even Electron Ions

1677 formula(e) evaluated with 5 results within limits (up to 50 closest results for each mass)

Elements Used:

C: 0-50 H: 0-100 N: 0-10 O: 0-25 S: 0-2

Minimum:			-1.5		
Maximum:	5.0	5.0	50.0		
Mass	Calc. Mass	mDa	PPM	DBE	Formula
368.1884	368.1875	0.9	2.4	15.5	C23 H22 N5
	368.1894	-1.0	-2.7	2.5	C11 H26 N7 O7
	368.1896	-1.2	-3.3	5.5	C19 H30 N O4 S
	368.1869	1.5	4.1	6.5	C15 H26 N7 O2 S
	368.1902	-1.8	-4.9	1.5	C12 H30 N7 O2 S2

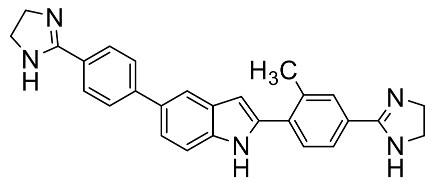

6b

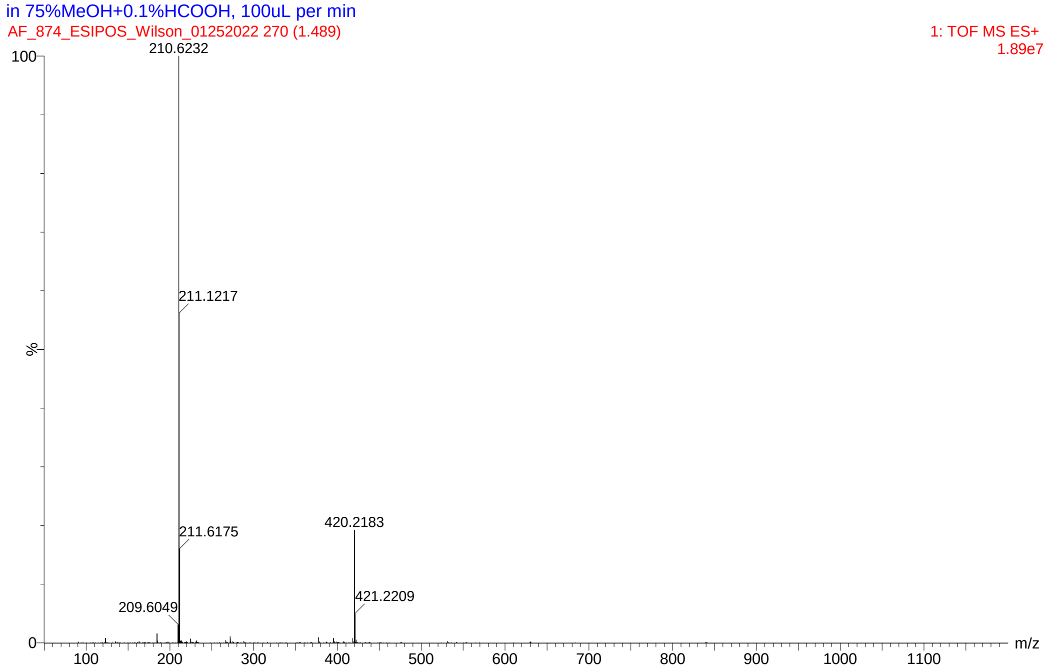
in 75%MeOH+0.1%HCOOH, 100uL per min
AF_874_ESIPOS_Wilson_01252022 270 (1.489)
1: TOF MS ES+
1.89e7
210.6232
211.1217
211.6175
209.6049
420.2183
421.2209
100
%
0
m/z
100
200
300
400
500
600
700
800
900
1000
1100

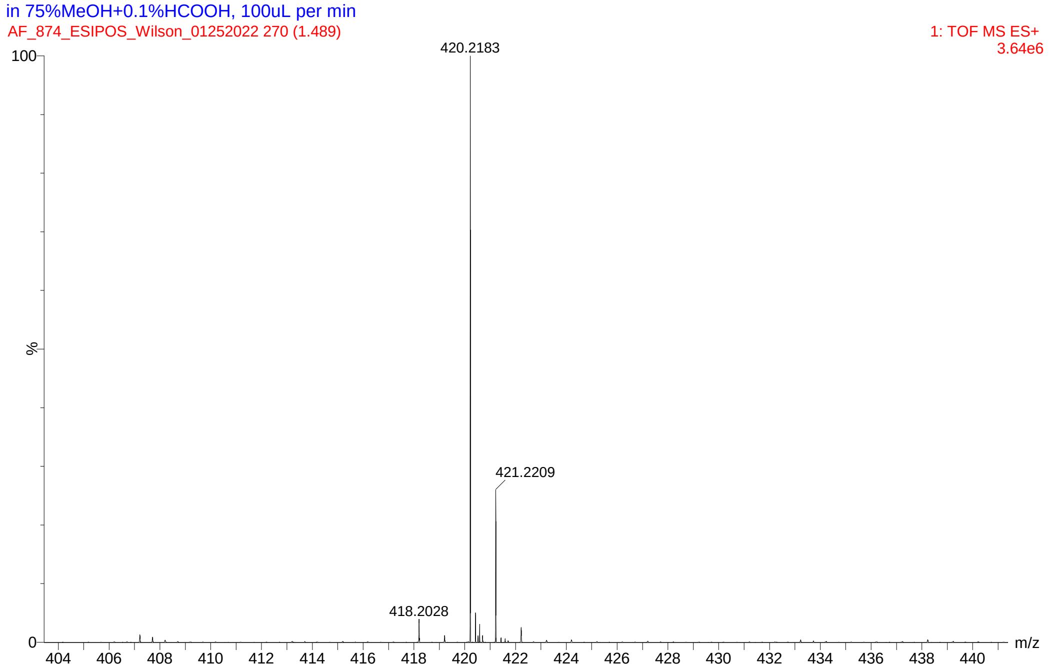
in 75%MeOH+0.1%HCOOH, 100uL per min
AF_874_ESIPOS_Wilson_01252022 270 (1.489)
1: TOF MS ES+
3.64e6
420.2183
421.2209
418.2028
100
%
0
m/z
404
406
408
410
412
414
416
418
420
422
424
426
428
430
432
434
436
438
440

Elemental Composition Report

Single Mass Analysis

Tolerance = 5.0 PPM / DBE: min = -1.5, max = 50.0

Element prediction: Off

Monoisotopic Mass, Even Electron Ions

2180 formula(e) evaluated with 8 results within limits (up to 50 closest results for each mass)

Elements Used:

C: 0-50 H: 0-100 N: 0-10 O: 0-25 S: 0-2

Minimum:			-1.5		
Maximum:	5.0	5.0	50.0		
Mass	Calc. Mass	mDa	PPM	DBE	Formula
420.2183	420.2182	0.1	0.2	8.5	C19 H30 N7 O2 S
	420.2188	-0.5	-1.2	17.5	C27 H26 N5
	420.2175	0.8	1.9	12.5	C26 H30 N O4
	420.2175	0.8	1.9	-0.5	C11 H34 N9 O4 S2
	420.2193	-1.0	-2.4	-0.5	C14 H34 N3 O11
	420.2168	1.5	3.6	3.5	C18 H34 N3 O6 S
	420.2166	1.7	4.0	0.5	C10 H30 N9 O9
	420.2202	-1.9	-4.5	-1.5	C15 H38 N3 O6 S2

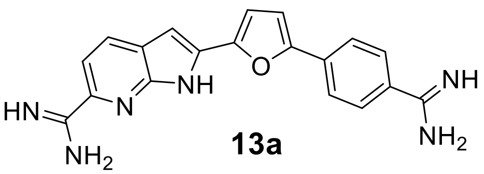
HN
NH2
N
NH
O
NH
NH2

13a

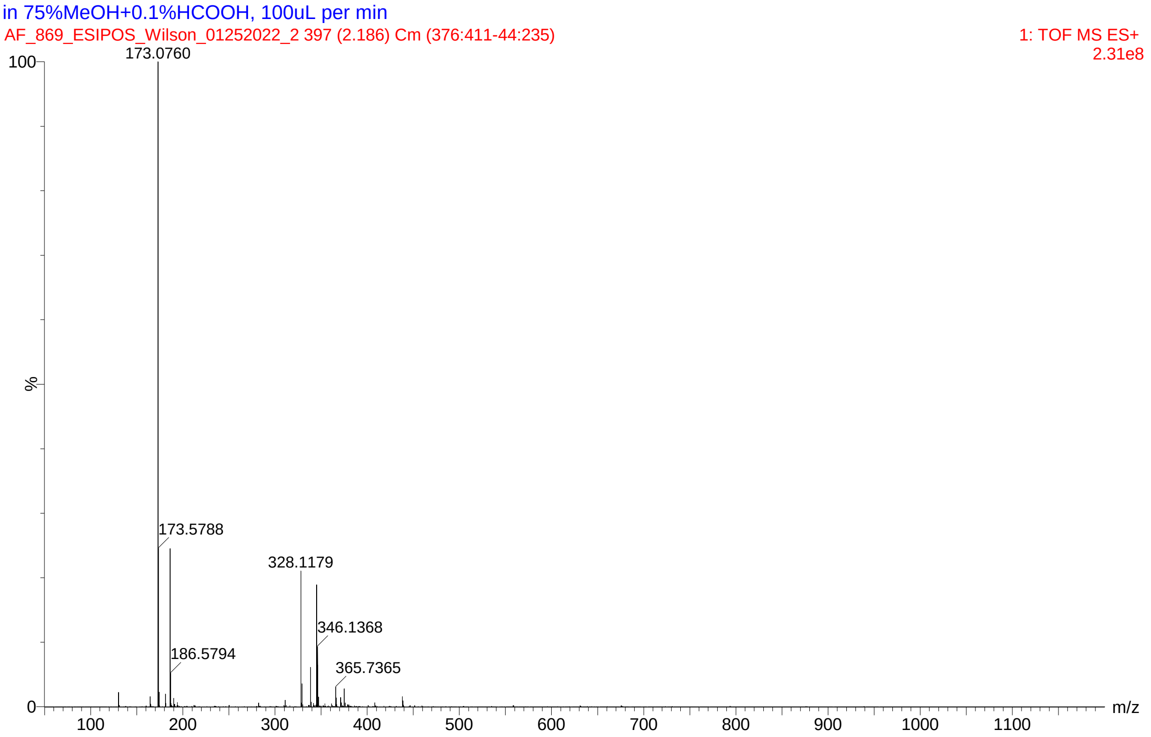
in 75%MeOH+0.1%HCOOH, 100uL per min
AF_869_ESIPOS_Wilson_01252022_2 397 (2.186) Cm (376:411-44:235)
1: TOF MS ES+
2.31e8
173.0760
173.5788
328.1179
346.1368
186.5794
365.7365
100
%
0
100
200
300
400
500
600
700
800
900
1000
1100
m/z

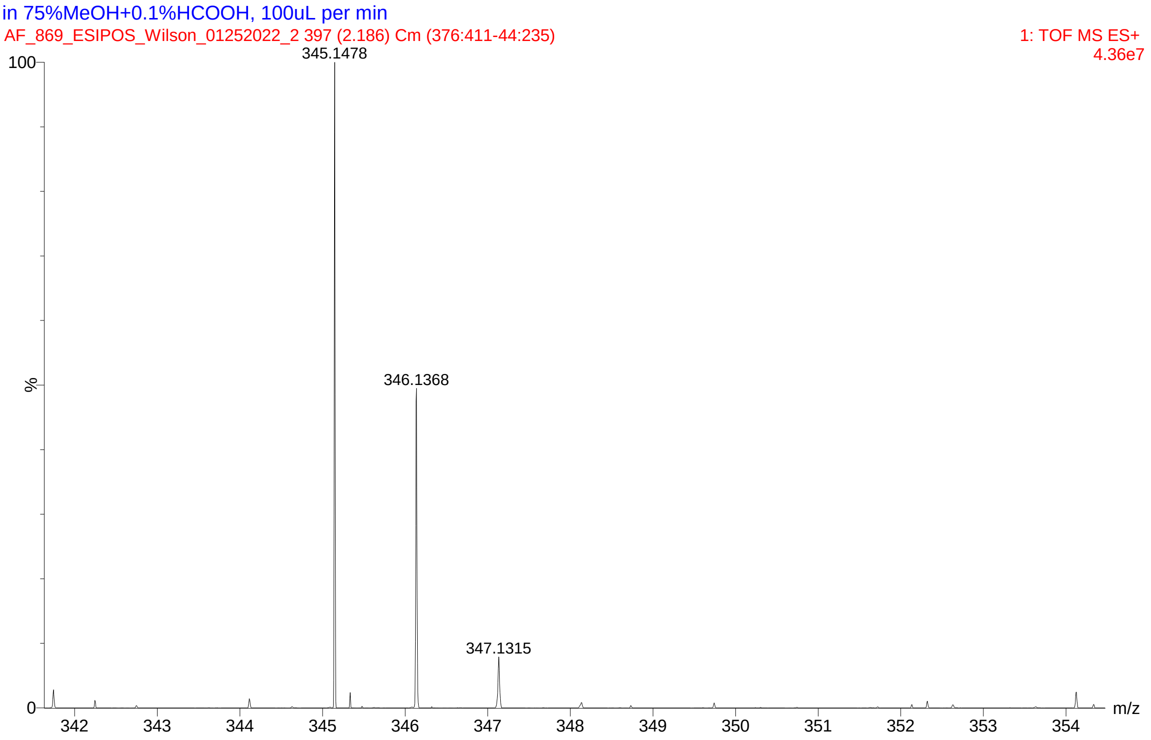
in 75%MeOH+0.1%HCOOH, 100uL per min
AF_869_ESIPOS_Wilson_01252022_2 397 (2.186) Cm (376:411-44:235)
1: TOF MS ES+
4.36e7
345.1478
346.1368
347.1315
100
%
0
342
343
344
345
346
347
348
349
350
351
352
353
354
m/z

Elemental Composition Report

Single Mass Analysis

Tolerance = 5.0 PPM / DBE: min = -1.5, max = 50.0

Element prediction: Off

Monoisotopic Mass, Even Electron Ions

594 formula(e) evaluated with 3 results within limits (up to 50 closest results for each mass)

Elements Used:

C: 0-50 H: 0-100 N: 0-10 O: 0-25

Minimum:			-1.5		
Maximum:	5.0	5.0	50.0		
Mass	Calc. Mass	mDa	PPM	DBE	Formula
345.1478	345.1482	-0.4	-1.2	1.5	C7 H21 N8 O8
	345.1491	-1.3	-3.8	13.5	C23 H21 O3
	345.1464	1.4	4.1	14.5	C19 H17 N6 O

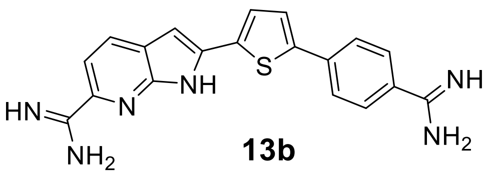
HN
N
NH
S
NH
NH$_2$
NH$_2$

13b

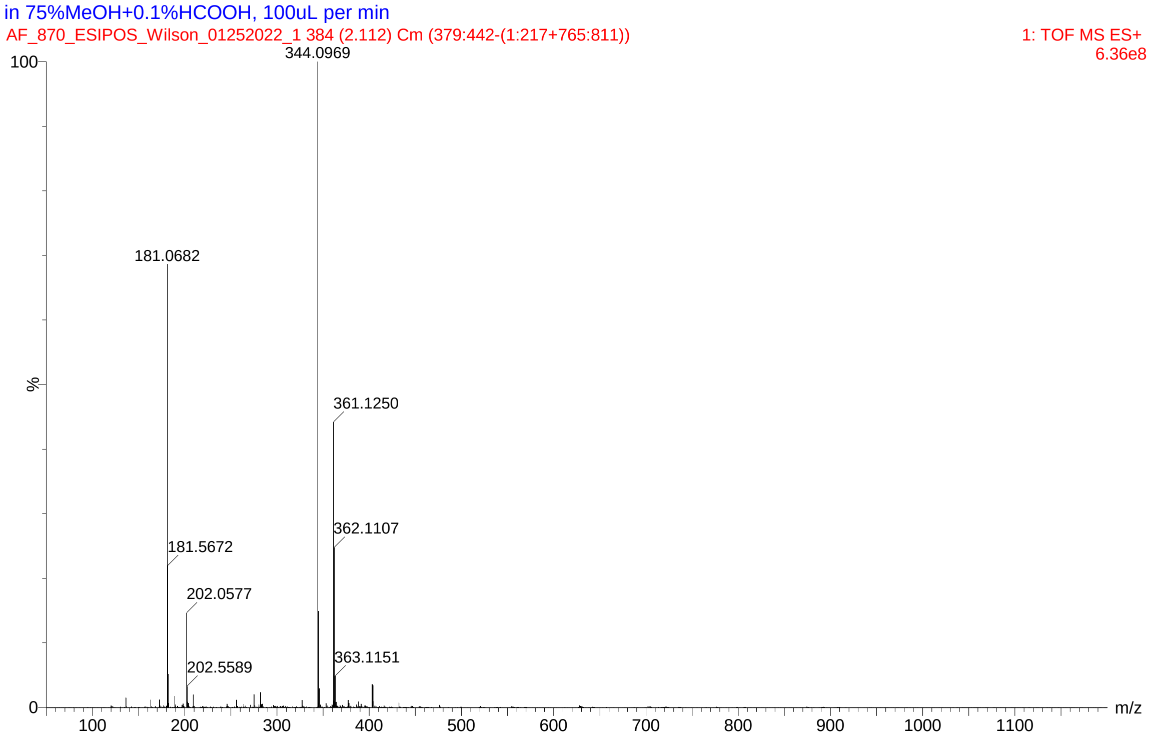
in 75%MeOH+0.1%HCOOH, 100uL per min
AF_870_ESIPOS_Wilson_01252022_1 384 (2.112) Cm (379:442-(1:217+765:811))
1: TOF MS ES+
6.36e8
344.0969
181.0682
361.1250
362.1107
181.5672
202.0577
202.5589
363.1151
%
m/z

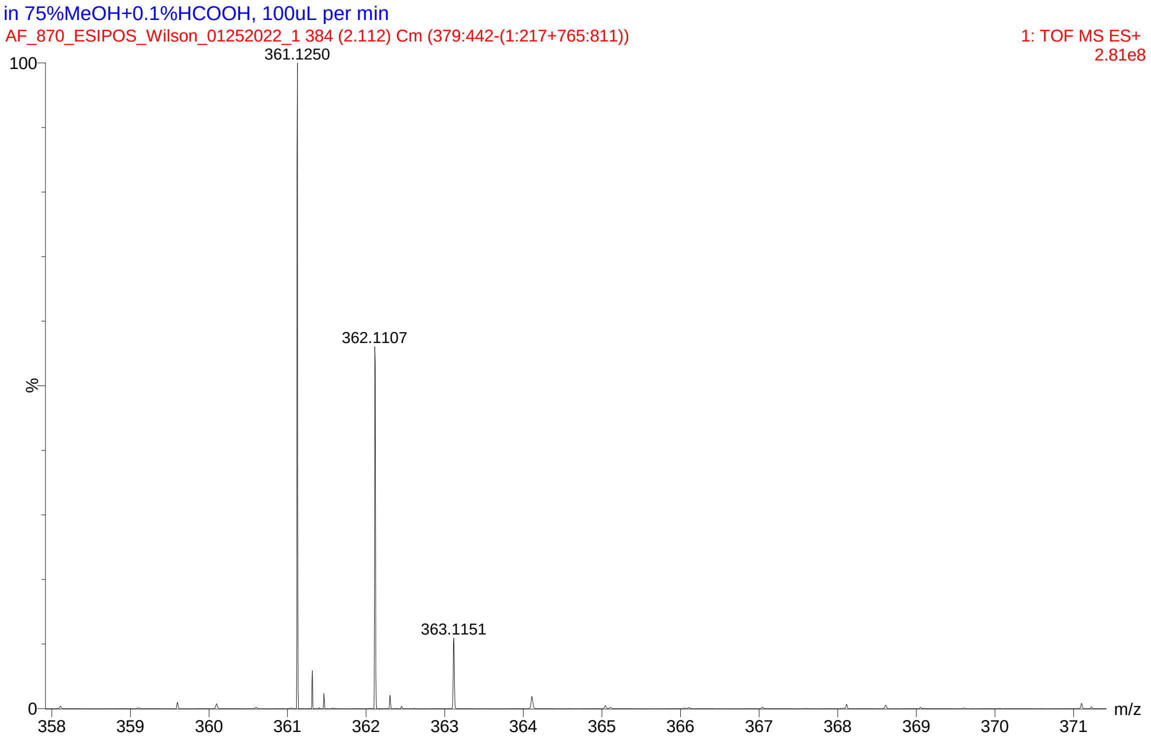
in 75%MeOH+0.1%HCOOH, 100uL per min
AF_870_ESIPOS_Wilson_01252022_1 384 (2.112) Cm (379:442-(1:217+765:811))
1: TOF MS ES+
2.81e8
361.1250
362.1107
363.1151
%
m/z

Elemental Composition Report

Single Mass Analysis

Tolerance = 5.0 PPM / DBE: min = -1.5, max = 50.0

Element prediction: Off

Monoisotopic Mass, Odd and Even Electron Ions

1639 formula(e) evaluated with 11 results within limits (up to 50 closest results for each mass)

Elements Used:

C: 0-50 H: 0-100 N: 0-10 O: 0-25 S: 0-2

Minimum:			-1.5		
Maximum:	5.0	5.0	50.0		
Mass	Calc. Mass	mDa	PPM	DBE	Formula
361.1250	361.1249	0.1	0.3	14.0	C21 H19 N3 O S
	361.1247	0.3	0.8	11.0	C13 H15 N9 O4
	361.1247	0.3	0.8	5.5	C14 H21 N2 O9
	361.1254	-0.4	-1.1	1.5	C7 H21 N8 O7 S
	361.1256	-0.6	-1.7	4.5	C15 H25 N2 O4 S2
	361.1242	0.8	2.2	5.0	C13 H23 N5 O3 S2
	361.1260	-1.0	-2.8	10.5	C15 H17 N6 O5
	361.1262	-1.2	-3.3	13.5	C23 H21 O2 S
	361.1235	1.5	4.2	14.5	C19 H17 N6 S
	361.1234	1.6	4.4	6.0	C12 H19 N5 O8
	361.1267	-1.7	-4.7	1.0	C9 H23 N5 O8 S

References:

1. Farahat, A.A.; Paliakov, E.; Kumar, A.; Barghash, A.-E.M.; Goda, F.E.; Eisa, H.M.; Wenzler, T.; Brun, R.; Liu, Y.; Wilson, W.D.; et al. Exploration of larger central ring linkers in furamidine analogues: Synthesis and evaluation of their DNA binding, antiparasitic and fluorescence properties. *Bioorg. Med. Chem.* **2011**, *19*, 2156-2167, doi:10.1016/j.bmc.2011.02.045.
2. Kumar, A.; Say, M.; Boykin, D.W. Synthesis of New Substituted 2-(Trimethylstannyl)indoles. *Synthesis* **2008**, *2008*, 707-710, doi:10.1055/s-2008-1032162.
3. CLSI. Performance Standards for Antimicrobial Susceptibility Testing; Fifteenth Informational Supplement. CLSI/NCCLS document M100-S15. **2005**.
4. Gulia, K.; Hassan, A.H.E.; Lenhard, J.R.; Farahat, A.A. Escaping ESKAPE resistance: in vitro and in silico studies of multifunctional carbamimidoyl-tethered indoles against antibiotic-resistant bacteria. *R. Soc. Open Sci.* **2023**, *10*, 230020, doi:doi:10.1098/rsos.230020.
